# Risk of new HIV diagnosis by intersecting migration, socioeconomic, and mental health vulnerabilities in the Netherlands: a nationwide analysis of the ATHENA cohort and Statistics Netherlands registry data

**DOI:** 10.1016/j.lanepe.2025.101508

**Published:** 2025-11-20

**Authors:** Vita W. Jongen, Anders Boyd, Patrizia Carrieri, Nina Schat, Selwyn H. Lowe, Rosan van Zoest, Marit G.A. van Vonderen, Jolanda Lammers, Mark Verhagen, Ard van Sighem, Marc van der Valk, ATHENA observational HIV cohort, F.J.B. Nellen, M.A. van Agtmael, M. Bomers, G.J. de Bree, S.E. Geerlings, A. Goorhuis, V.C. Harris, J.W. Hovius, B. Lemkes, E.J.G. Peters, T. van der Poll, J.M. Prins, K.C.E. Sigaloff, V. Spoorenberg, M. van der Valk, M. van Vugt, W.J. Wiersinga, F.W.M.N. Wit, C. Bruins, J. van Eden, I.J. Hylkema-van den Bout, L.M. Laan, F.J.J. Pijnappel, S.Y. Smalhout, M.E. Spelbrink, A.M. Weijsenfeld, N.K.T. Back, R. van Houdt, M. Jonges, S. Jurriaans, F. van someren Gréve, M.R.A. Welkers, K.C. Wolthers, M. van der Kuip, D. Pajkrt, F.M. Hessing, A.M. Weijsenfeld, M. van den Berge, A. Stegeman, S. Baas, L. Hage de Looff, A. van Arkel, J. Stohr, B. Wintermans, M.J.H. Pronk, H.S.M. Ammerlaan, E.S. de Munnik, S. Phaf, B. Deiman, V. Scharnhorst, M.C.A. Wegdam, J. Nellen, A. van Eeden, E. Hoornenborg, S. de Stoppelaar, H. Berends, L.J.M. Elsenburg, H. Nobel, F. van Someren Gréve, M. Welkers, K. Wolthers, N. Back, S. Jurriaans, M.E.E. van Kasteren, M.A.H. Berrevoets, A.E. Brouwer, A. Adams, B.A.F.M. de Kruijf-van de Wiel, M. Pauwels-van Rijkevoorsel, J.L. Murck, C. Rokx, A.A. Anas, H.I. Bax, E.C.M. van Gorp, M. de Mendonça Melo, E. van Nood, J.L. Nouwen, B.J.A. Rijnders, C.A.M. Schurink, L. Slobbe, T.E.M.S. de Vries-Sluijs, N. Bassant, J.E.A. van Beek, M. Vriesde, L.M. van Zonneveld, J. de Groot, J.J.A. van Kampen, M.P.G. Koopmans, P.L.A. Fraaij, A.M.C. van Rossum, C.L. Vermont, L.C. van der Knaap, J. Branger, R.A. Douma, A.S. Cents-Bosma, M.A. Mulder, E.F. Schippers, C. de Bree, C. van Nieuwkoop, J. Geilings, A. van Overeem, G. van der Hut, N.D. van Burgel, E.M.S. Leyten, L.B.S. Gelinck, F. Mollema, M. Langbein, G.S. Wildenbeest, T. Nguyen, B. Hafkamp, J.W. Bouwhuis, A.J.J. Lammers, A.G.W. van Hulzen, S. Kraan, S.B. Debast, G.H.J. Wagenvoort, A.H.E. Roukens, M.G.J. de Boer, H. Jolink, M.M.C. Lambregts, H. Scheper, A. Metselaar, D. van der Sluis, S.A. Boers, E.C.J. Claas, E. Wessels, J.G. den Hollander, R. El Moussaoui, K. Pogany, C.J. Brouwer, D. Heida-Peters, E. Mulder, J.V. Smit, D. Struik-Kalkman, T. van Niekerk, C. van Tienen, S.H. Lowe, A.M.L. Oude Lashof, D. Posthouwer, A. Stoop, M.E. van Wolfswinkel, R.P. Ackens, M. Elasri, K. Houben-Pintaric, J. Schippers, T.R.A. Havenith, I.H.M. van Loo, M.G.A. van Vonderen, L.M. Kampschreur, S.E. van Roeden, M.C. van Broekhuizen, A. Al Moujahid, G.J. Kootstra, C.E. Delsing, M. van der Burg-van de Plas, L. Scheiberlich, W. Kortmann, G. van Twillert, R. Renckens, J. Wagenaar, D. Ruiter-Pronk, B. Stander, J.W.T. Cohen Stuart, M. Hoogewerf, W. Rozemeijer, J.C. Sinnige, K. Brinkman, G.E.L. van den Berk, K.D. Lettinga, M. de Regt, W.E.M. Schouten, J.E. Stalenhoef, S.M.E. Vrouenraets, H. Blaauw, G.F. Geerders, M.J. Kleene, M. Knapen, M. Kok, I.B. van der Meché, A.J.M. Toonen, S. Wijnands, E. Wttewaal, D. Kwa, T.J.W. van de Laar, R. van Crevel, K. van Aerde, R.J.W. Arts, S.S.V. Henriet, H.J.M. ter Hofstede, J. Hoogerwerf, O. Richel, K. Stol, M. Albers, K.J.T. Grintjes-Huisman, M. de Haan, M. Marneef, M. McCall, J. Rahamat-Langendoen, E. Ruizendaal, D. Burger, E.H. Gisolf, M. Claassen, R.J. Hassing, G. ter Beest, P.H.M. van Bentum, Y. Neijland, M. Valette, C.M.A. Swanink, M. Klein Velderman, S.F.L. van Lelyveld, R. Soetekouw, L.M.M. van der Prijt, J. van der Swaluw, J.S. Kalpoe, A. Wagemakers, A. Vahidnia, F.N. Lauw, D.W.M. Verhagen, M. van Wijk, W.F.W. Bierman, M. Bakker, J. Kleinnijenhuis, E. Kloeze, A. Middel, D.F. Postma, Y. Stienstra, M. Wouthuyzen-Bakker, A. Boonstra, M.M.M. Maerman, D.A. de Weerd, M. Knoester, C.C. van Leer-Buter, H.G.M. Niesters, X.W. Zhou, B.R. Brandsema, A.R. Verhage, N. van der Woude, M. Knoester, C.C. van Leer-Buter, H.G.M. Niesters, X.W. Zhou, T. Mudrikova, R.E. Barth, A.H.W. Bruns, P.M. Ellerbroek, M.P.M. Hensgens, J.J. Oosterheert, E.M. Schadd, A. Verbon, B.J. van Welzen, B.M.G. Griffioen-van Santen, L. van de Koolwijk, I. de Kroon, F.M. Verduyn Lunel, A.M.J. Wensing, Y.G.T. Loeffen, T.F.W. Wolfs, M. Kok, F.M. Verduyn Lunel, A.M.J. Wensing, E.O.W. Rooijakkers, D. van de Wetering, A. Alberto, M. van der Valk, S. Zaheri, A.C. Boyd, D.O. Bezemer, V.W. Jongen, A.I. van Sighem, C. Smit, F.W.M.N. Wit, M.M.J. Hillebregt, T.J. Woudstra, T. Rutkens, D. Bergsma, J.M. Grolleman, L.E. Koster, K.J. Lelivelt, S.T. van Loenen, M.J.C. Schoorl, K.M. Visser, K.J. Lelivelt, K.M. Visser, M. van den Akker, O.M. Akpomukai, R. Alexander, Y.M. Bakker, L. Bastos Sales, A.el Berkaoui, M. Bezemer-Goedhart, C.B.J. Bon, E.A. Djoechro, I. el Hammoud, M.R. Khouw, C.R.E. Lodewijk, E.G.A. Lucas, S. van Meerveld-Derks, M.A. van Montfoort, H.W. Mulder, L. Munjishvili, C.M.J. Ree, R. Regtop, A.F. van Rijk, Y.M.C. Ruijs-Tiggelman, P.P. Schnörr, R. van Veen, W.H.G. van Vliet-Klein Gunnewiek, E.C.M. Witte, D. Bergsma, Y.M.C. Ruijs-Tiggelman

**Affiliations:** a Stichting HIV Monitoring, Amsterdam, the Netherlands; b Department of Infectious Diseases, Public Health Service Amsterdam, the Netherlands; c Amsterdam University Medical Center, Department of Infectious Diseases, University of Amsterdam, Amsterdam Infection & Immunity Institute, Amsterdam, the Netherlands; d Aix Marseille Univ, INSERM, IRD, SESSTIM, Sciences Économiques & Sociales de la Santé & Traitement de L'information Médicale, ISSPAM, Marseille, France; e Amsterdam Health & Technology Institute, Amsterdam, the Netherlands; f Department of Medical Microbiology, Infectious Diseases and Infection Prevention (MMI) and Department of Internal Medicine, Maastricht University Medical Center, Maastricht, the Netherlands; g Department of Internal Medicine, Frisius Medical Center location Leeuwarden, Leeuwarden, Netherlands; h Department of Internal Medicine, Isala Hospital, Zwolle, the Netherlands

**Keywords:** HIV, Healthcare disparities, Socioeconomic factors, Health inequalities, Demography

## Abstract

**Background:**

To further reduce new HIV diagnoses in the Netherlands, individual and structural barriers hindering prevention must be addressed. We aimed to estimate the disproportional burden of new HIV diagnoses and explore how intersecting socio-demographic, socio-economic, and health-related factors jointly influence the risk of a new HIV diagnosis.

**Methods:**

We combined data from the ATHENA cohort, an ongoing nationwide HIV cohort, with registry data from Statistics Netherlands. We selected individuals with a new HIV diagnosis between 1 January 2012 and 31 December 2023 and matched them to individuals from the general population. We assessed determinants of a new HIV diagnosis using a multivariable generalized linear model. We used Multilevel Analysis of Individual Heterogeneity and Discriminatory Accuracy (MAIHDA) to quantify the joint and individual contribution of intersecting variables.

**Findings:**

6055 men and 1020 women were newly diagnosed with HIV. Having a migration background and a low to middle income or income below the poverty line was associated with a higher risk of a new HIV diagnosis for both men (low to middle: adjusted odd ratio (aOR) = 1.24, 95% confidence interval (CI) = 1.17–1.31; below the poverty line: aOR = 1.75, 95% CI = 1.62–1.89) and women (low to middle: aOR = 2.49, 95% CI = 2.05–3.01; below the poverty line: aOR = 4.71, 95% CI = 3.80–5.83). Use of mental health care (aOR = 1.14, 95% CI = 1.01–1.27) or antidepressants (aOR = 1.66, 95% CI = 1.50–1.84) also increased the risk among men; while receiving social welfare (aOR = 1.39, 95% CI = 1.15–1.67) and use of antipsychotic medication (aOR = 1.66, 95% CI = 1.21–2.28) increased the risk among women. Of all intersections identified in MAIHDA, men with a first-generation migration background, income below the poverty line, and who used antidepressants had the highest predicted probability of an HIV diagnosis (0.036%, 95% confidence interval (CI) = 0.025–0.052). Women with a first-generation background, income below the poverty line, who received social welfare, and who used antipsychotic medication had the highest predicted risk (0.019%, 95% CI = 0.011–0.035).

**Interpretation:**

A disproportionally higher burden of a new HIV diagnosis was observed for individuals with a migration background and economic and mental health vulnerabilities. HIV prevention and testing need to be reinforced in these groups.

**Funding:**

10.13039/501100002999Dutch Ministry of Health, Welfare and Sport; TKI Health Holland.

**Research in context**

**Evidence before this study**

It is currently unknown how the HIV epidemic is shaped by socio-demographic and -economic differences in the Netherlands, while this information is important to develop targeted intervention strategies or recommendations. We searched PubMed on 9 May 2025 with no language or date restrictions, using the terms [“HIV”] AND [“burden”] AND [“inequalities”] AND [“Europe”] OR [“Netherlands”]. Previous studies have found large inequalities in certain aspects of the HIV epidemic within European countries. Lower awareness of HIV and less frequent testing are more common in individuals with a lower education level and socio-economic status. Similarly, socio-economically disadvantaged individuals experience poorer access to biomedical HIV prevention tools. These previous studies have rarely focused on people with newly diagnosed HIV, are limited in the types of socioeconomic data included and might have strong selection biases. We also found no papers describing inequalities of those with newly diagnosed HIV in the Netherlands.

**Added value of this study**

Using population-based data from the ATHENA cohort, comprising data from over 97% of all individuals with HIV in care in the Netherlands, and non-public registry data from Statistics Netherlands, we compared extensive socio-demographic, socio-economic, and health-related determinants of individuals with a new HIV diagnosis to the general Dutch population aged 18 years or older. We found that higher risk of new HIV diagnoses was observed in those with several sociodemographic (i.e., first- or second-generation migration background) and socioeconomic factors (i.e., low to middle income or income below the poverty line and social welfare), as well as in those utilising specific mental health services. Importantly, there was clear intersectionality between these variables, explaining some of the risk for new HIV diagnoses.

**Implications of all the available evidence**

New HIV diagnoses are occurring disproportionately higher in individuals with lower socioeconomic status, those in difficult living situations, and those needing mental health care; all hallmarks of social deprivation. These findings allow us to go beyond key populations and focus on more specific subgroups who could benefit from improved HIV prevention and treatment. Reducing health inequalities is likely needed to move towards zero new HIV infections in the Netherlands.

## Introduction

HIV prevention and treatment options have improved enormously since the start of the HIV epidemic, which together have provided drastic decreases in new HIV acquisitions. Nevertheless, inequalities in the burden of HIV infections persist and remain a major barrier to the global HIV response.^1^ While sex-inequalities in HIV infections are substantial (with more HIV diagnoses among men in most Western-European countries),^1^^,^^2^ other socio-demographic or -economic factors may underly these inequalities. Previous studies have shown that lower awareness of HIV and less frequent HIV testing are more common in individuals with a lower education level and socio-economic status.^1^^,^^3^^,^^4^ Moreover, these inequalities are also apparent after HIV acquisition, as socio-economically disadvantaged individuals face poorer access to treatment and worse HIV-related outcomes.^1^^,^^5^, ^6^, ^7^

In the Netherlands, the HIV care continuum has almost reached the 95-95-95 UNAIDS targets in recent years; however, these targets have not yet been met for women and cisgender heterosexual men.^2^ Dutch health care services are universally accessible, STI and HIV testing is free for specific populations [including men who have sex with men (MSM) and transgender persons], and HIV prevention services, namely PrEP, are available at reduced costs. Documented migrants have equal access to these services, while undocumented migrants or uninsured individuals are required to pay out-of-pocket for most healthcare services, unless certain financial coverage is obtained by a health professional (as is the case with HIV-related care) or in the case of urgent care.^8^ Undocumented migrants do, however, have access to free and anonymous HIV and STI screening. Despite these services, there is no longer a decline in the number of new HIV diagnoses in recent years with 494 individuals newly diagnosed in 2023.^2^ This stagnation likely reflects individual, interpersonal, and structural barriers that hinder prevention.

Structural determinants—including immigration status, language barriers, stigma, discrimination in healthcare, and socio-economic precarity—contribute to delayed diagnosis, reduced access to care, and suboptimal treatment outcomes.^9^ For instance, undocumented migrants may avoid seeking care due to fear of deportation or lack of information about social support.^10^^,^^11^ Stigma and racial discrimination also continue to undermine mental health and retention in HIV care.^11^

To address these inequities, it is essential to apply recent frameworks that incorporate structural determinants and their intersectionalities.^12^^,^^13^ These types of frameworks use empirical data with which the social burdens of individuals with newly diagnosed HIV are compared to those in the general population. However, such an analysis has yet to be conducted in the Netherlands. We then aimed to estimate the disproportionate burden of new HIV diagnoses and explore how intersecting socio-demographic, socio-economic, and health-related factors jointly influence the risk of a new HIV diagnosis using data from the ATHENA national HIV cohort and registry data from Statistics Netherlands. This approach provides a more nuanced understanding of structural inequalities in the risk of HIV, which can inform targeted screening strategies and policy recommendations aimed at addressing health inequities more effectively.

## Methods

### Study design and data collection

We conducted a secondary analysis leveraging individual data from the ATHENA cohort and non-public microdata from Statistics Netherlands.

Briefly, HIV care in the Netherlands is provided by 23 designated treatment centres. The HIV Monitoring Foundation [Stichting hiv monitoring (SHM)] is tasked by the Dutch Ministry of Healthcare, Welfare and Sports to monitor and report on all aspects of HIV care for people with HIV in the Netherlands. Data collection was initiated in 1998 and data are prospectively collected in the ATHENA (AIDS Therapy Evaluation in the Netherlands) cohort, which represents over 97% of all people with HIV in care in the Netherlands.^14^

Statistics Netherlands (*Centraal Bureau voor de Statistiek*, CBS) is an independent organization that collects, processes and publishes reliable statistical data on residents of the Netherlands. The Statistics Netherlands Act constitutes the legal basis for Statistics Netherlands, and Statistics Netherlands is adherent to the European Union's General Data Protection Regulation.

Data from the ATHENA cohort were uploaded to the secure Remote Access environment hosted by Statistics Netherlands. Data linkage between data from ATHENA and microdata from Statistics Netherlands was facilitated by Statistics Netherlands using a probabilistic approach based on individual date of birth, first four digits of the postal code of last known residence and sex at birth. Statistics Netherlands performed exact matching. Any linkage error would be the result of measurement error (e.g., mis-registered data in one of the data registries) or the inability to perform exact matching (e.g., two people with the exact same date of birth and sex registered at a single postal code). Any data with linkage error were discarded.

### Study population

We selected all individuals in the ATHENA cohort who were 18 years or older and newly diagnosed with HIV in the Netherlands between 1 January 2012 and 31 December 2023. Individuals who migrated to the Netherlands with a known pre-migration HIV diagnosis were excluded. We also excluded individuals identifying as transgender as few were included in the ATHENA cohort and the risk of identification was deemed non-negligible. We selected all individuals from the non-public microdata made available by Statistics Netherlands who were aged 18 years and older. We matched each individual newly diagnosed with HIV to all individuals from the general population with the same year of birth, gender, and calendar year of HIV diagnosis without replacement. Matching was performed using coarsened exact matching with the *cem* package in R.

### Study variables

At enrolment into the ATHENA cohort, the following demographic information was collected: year of birth, country of birth, sex assigned at birth, gender identity (if different from sex at birth), and most likely transmission route of HIV. As part of the ATHENA cohort, additional information about the date of HIV diagnosis was retrieved from the referral letter provided by the general practitioner or Centre for Sexual Health, from health records in the HIV treatment centre, or self-reported if no documentation was available.

Statistics Netherlands provided detailed individual-based socio-demographic and socio-economic information, including education level, migration background, employment status, household composition, household income, and use of social welfare. Household income was defined according to the social minimum (the amount of financial resources required to achieve a minimally acceptable lifestyle). The social minimum is determined and adjusted bi-annually by the Ministry of Social Affairs and Employment.^15^ An individual's income was categorized as below the poverty line if their household income was <120% of the social minimum, which determines if an individual gets benefits in the Netherlands. Missing income data at the time of HIV diagnosis were imputed using the closest data from within 3 calendar years of HIV diagnosis (for individuals with newly diagnosed HIV) or the matched year (for individuals from the general population).

Additionally, Statistics Netherlands provided information on health expenditure paid through universal health insurance in the Netherlands. Every health expenditure is assigned a Diagnostic-Treatment-Classification (DTC), which reflects a diagnosis or treatment. We included data on use of the Long-term Care Act (defined as declared costs >0 Euro associated with this Act), mental health care (defined as declared costs >0 Euro for basic or specialized mental health care), use of antipsychotics, and use of antidepressants. The Long-term Care Act entails care with stay and care at home, elderly care, psychiatric care, care during chronic illness, and care for individuals with a disability.

Socio-demographic and -economic and health related information was based on data registered at the end of the previous calendar year (i.e., 31 December of the year prior to HIV diagnosis).

### Statistical analysis

All analyses were stratified on gender given their vastly differing HIV epidemics in the Netherlands.^2^ Missing values were analyzed as a separate category for all variables, except for income. Since missingness of income was non-informative for measured covariates and a substantial percentage of income data was missing, we decided not to make any inference on a missing category and excluded observations with missing income data. We first modelled the probability of having an HIV diagnosis using a generalized linear model with logit link and quasibinomial family (to account for overdispersion). We weighted this model by a factor obtained from coarsened exact matching to equalize the number of individuals with and without HIV acquisition within matched strata. We assessed separate covariates to obtain univariable odds ratios (OR) and their 95% confidence intervals (CI). We excluded any variables with excessive missingness or combinations with strong multicollinearity as determined from Cramér's V or from previous research (i.e., education and income, while leaving income in the model). All variables that were statistically significant in univariable analysis and did not show multicollinearity were included in the initial multivariable model.

We then conducted a Multilevel Analysis of Individual Heterogeneity and Discriminatory Accuracy (MAIHDA).^16^ MAIHDA is a multilevel modelling approach designed to quantify both the joint and individual contributions of intersecting variables (e.g., income, migration background and mental health) to an outcome, while accounting for between-group heterogeneity. We used the variables identified through multivariable logistic regression analysis to define strata, with each unique combination of variables forming a single stratum. We first created a baseline model in which only a random intercept for the strata was included. From this model, we estimated the variance partition coefficient (VPC) as a measure of the extent to which differences in the probability of HIV diagnoses could be attributed to stratum-level factors. In a second model, we added age and all variables used to define the strata as fixed effects to estimate their individual contributions and to assess whether between-stratum variation remained after accounting for these main effects. Age was included as a fixed effect to minimize residual confounding after matching. We then derived predicted probabilities of an HIV diagnosis for each stratum based on the fixed and random effects from the final MAIHDA model, allowing us to identify intersectional groups with higher or lower odds of an HIV diagnosis.

The results presented are based on calculations carried out by the SHM using non-public microdata from Statistics Netherlands (CBS) and Vektis C.V. Statistical analyses were performed using Stata (v19.5, StataCorp, College Station, TX, USA) and R (version 4.2.1, Vienna, Austria).

### Ethics approval

People entering HIV care receive written material about participation in the ATHENA cohort, after which they are asked to consent verbally to the use of their routinely collected medical data for research and monitoring (i.e., an “opt-in” procedure). Participants can withdraw their consent at any time. Data collection was approved by boards of all participating centres. Routinely collected data were used for this analysis and therefore no additional review or consent was required.

Participants from the ATHENA cohort provided consent for use of their data for data linkage purposes and information about active data linkages is available on the SHM website. The Remote Access environment is only available for researchers authorized by SHM and Statistics Netherlands. All output from the Remote Access environment is independently verified by Statistics Netherlands to ensure data cannot be traced back to individuals.

### Role of the funder

The funder of the study had no role in study design, data collection, data analysis, data interpretation, or writing of the report.

## Results

Between 1 January 2012 and 31 December 2023, 6055 men and 1020 women were newly diagnosed with HIV. 2437 (40%) men and 551 (54%) women were diagnosed with late or advanced stage HIV. These individuals were matched to 75,774,149 men and 66,819,245 women from the general population (Table 1). 2466 men and 659 women with HIV had a first or second migration background and 1388 men and 455 women with HIV had an income below the poverty line.

**Table 1:** Socio-demographic and -economic characteristics of individuals newly diagnosed with HIV and the general population.

	Men		Women	
	With HIV N = 6055	General population N = 75,774,149	With HIV N = 1020	General population N = 66,819,245
	n (%)	n (%)	n (%)	n (%)
**Age**				
<25 years	516 (8.5%)	8,882,666 (12%)	108 (11%)	8,162,257 (12%)
25–49 years	3868 (64%)	33,079,503 (44%)	650 (64%)	32,635,388 (49%)
≥50 years	1671 (28%)	33,811,980 (45%)	262 (26%)	26,021,600 (39%)
**Migration background**				
None	3589 (59%)	58,640,327 (77%)	361 (35%)	50,355,492 (75%)
First generation	1839 (30%)	10,473,462 (14%)	566 (55%)	10,531,077 (16%)
Second generation	627 (10%)	6,660,360 (8.8%)	93 (9.1%)	5,932,676 (8.9%)
**Education**				
Primary	832 (14%)	9,804,482 (13%)	232 (23%)	8,633,655 (13%)
Secondary	1529 (25%)	19,272,235 (25%)	214 (21%)	17,242,958 (26%)
College/University	967 (16%)	13,901,692 (18%)	87 (8.5%)	14,374,900 (22%)
Missing	2727 (45%)	32,795,740 (43%)	487 (48%)	26,567,732 (40%)
**Income**				
Below the poverty line^a^	1388 (23%)	9,197,382 (12%)	455 (45%)	9,390,488 (14%)
Middle-low	2631 (43%)	31,758,122 (42%)	424 (42%)	27,626,848 (41%)
High	2036 (34%)	34,818,645 (46%)	141 (14%)	29,801,909 (45%)
**Received social welfare**	491 (8.1%)	3,017,177 (4.0%)	209 (20%)	3,457,033 (5.2%)
**Single person household**^b^				
No	2599 (43%)	55,502,179 (73%)	354 (35%)	48,006,250 (72%)
Yes	3017 (50%)	20,031,289 (26%)	543 (53%)	18,621,338 (28%)
Missing	439 (7.3%)	240,681 (0.3%)	123 (12%)	191,657 (0.3%)
**Used mental health care**^c^	437 (7.2%)	3,549,901 (4.7%)	90 (8.8%)	4,662,969 (7.0%)
**Used antidepressants**^d^	488 (8.1%)	3,907,013 (5.2%)	119 (12%)	6,198,584 (9.3%)
**Used antipsychotics**^d^	164 (2.7%)	1,580,540 (2.1%)	49 (4.8%)	1,395,085 (2.1%)

a Income below the poverty line is defined as a household income <120% of the social minimum (the minimal amount of financial resources required to achieve a minimally acceptable lifestyle). The social minimum is determined and adjusted bi-annually by the Ministry of Social Affairs and Employment (https://www.uwv.nl/nl/toeslag/sociaal-minimum).

b Includes single-parent households.

c Defined as declared cost (>0 euro) for mental health care.

d Use of medication for depression (ATC code N06A) or psychosis (ATC code N05A).

Results from the univariable logistic regression analysis are shown in Supplementary Table S1. For men, in multivariable analysis, having a first or second generation migration background, a low to middle income or income below the poverty line, mental health care use, and the use of antidepressants were all associated with higher odds of newly diagnosed HIV (Table 2). In contrast, use of antipsychotic medication was associated with a lower odds of newly diagnosed HIV. For women, having a first or second generation migration background, a low to middle income or income below the poverty line, receiving social welfare, and the use of antipsychotic medication were all associated with increased odds of newly diagnosed HIV.

**Table 2:** Determinants of an HIV diagnosis. Results from multivariable logistic regression.

	Men		Women	
	aOR	95% CI	aOR	95% CI
**Migration background**				
None	REF		REF	
First generation	2.21	2.08–2.35	4.48	3.87–5.19
Second generation	1.33	1.22–1.44	1.65	1.31–2.07
**Income**				
High	REF		REF	
Middle-low	1.24	1.17–1.31	2.49	2.05–3.01
Below the poverty line^a^	1.75	1.62–1.89	4.71	3.80–5.83
**Received social welfare**				
No			REF	
Yes			1.39	1.15–1.67
**Used mental health care**^b^				
No	REF			
Yes	1.14	1.01–1.27		
**Used antidepressants**^c^				
No	REF		REF	
Yes	1.66	1.50–1.84	1.23	0.99–1.52
**Used anti-psychotic medication**^c^				
No	REF		REF	
Yes	0.79	0.66–0.94	1.66	1.21–2.28

a Income below the poverty line is defined as a household income <120% of the social minimum (the minimal amount of financial resources required to achieve a minimally acceptable lifestyle). The social minimum is determined and adjusted bi-annually by the Ministry of Social Affairs and Employment (https://www.uwv.nl/nl/toeslag/sociaal-minimum).

b Defined as declared cost (>0 euro) for mental health care.

c Use of medication for depression (ATC code N06A) or psychosis (ATC code N05A).

In the MAIHDA, we generated intersectional strata for men and women separately based on the results of the final multivariable models. For men, strata were defined by combining categories of migration background (none, first generation, second generation), income (high, low to middle, below poverty line), mental health care use (yes/no), antidepressant use (yes/no), and antipsychotic medication use (yes/no). For women, strata were combined based on migration background, income, receiving social welfare (yes/no), use of antidepressants, and use of antipsychotic medication. This approach yielded 72 strata for both men and women. As age was included as an additional variable in the multivariable models, predictions were generated for each age category within the strata, resulting in a total of 216 predicted combinations. Number of observations for these 216 combinations can be found in Supplementary Table S2.

**Fig. 1:**
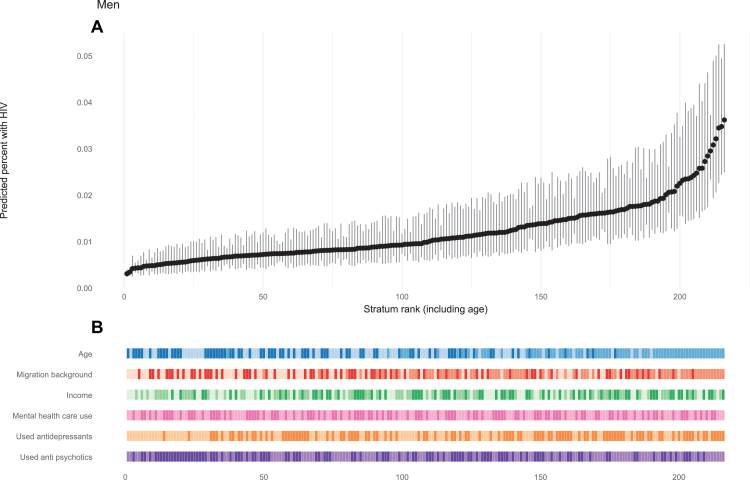
**Predicted probabilities of a new HIV diagnosis for each stratum among men**. A) Predicted probabilities, B) Characteristics associated with each stratum. In B) Color coding indicates categories for each variable. Age: dark blue = ≥50 years, medium blue = 25–49 years, light blue <25 years. Migration background: light red = no migration background, medium red = first generation migration background, dark red = second generation migration background. Income: dark green = below poverty line, medium green = low to middle income, light green = high income. For the other variables (mental health care, antidepressant use, antipsychotic use): darker shades = used, lighter shades = not used.

**Fig. 2:**
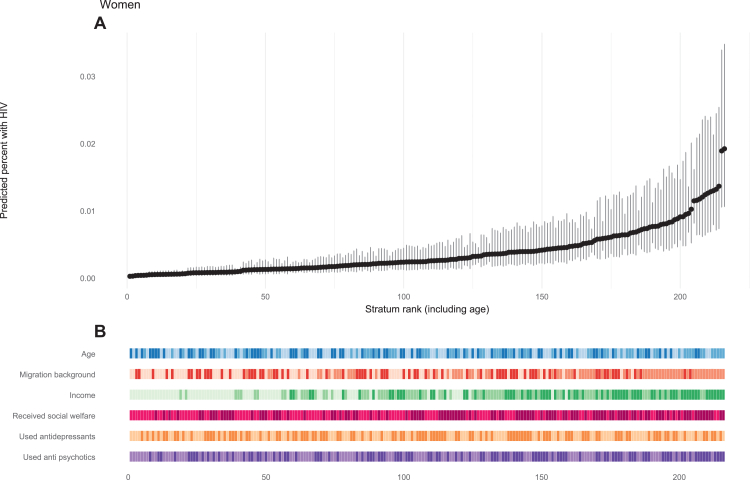
**Predicted probabilities of a new HIV diagnosis for each stratum among women**. A) Predicted probabilities, B) Characteristics associated with each stratum. In B) Color coding indicates categories for each variable. Age: dark blue = ≥50 years, medium blue = 25–49 years, light blue <25 years. Migration background: light red = no migration background, medium red = first generation migration background, dark red = second generation migration background. Income: dark green = below poverty line, medium green = low to middle income, light green = high income. For the other variables (received social welfare, antidepressant use, antipsychotic use): darker shades = received/used, lighter shades = not received/used.

In the baseline model including only a random intercept for stratum, the VPC indicated that approximately 4.6% of the variance in new HIV diagnoses among men was attributable to differences between strata; for women the VPC was 20.4% (Table 3). When fixed effects were added to the models, the proportional change in variance (PVC) was 66.6% for men and 92.5% for women, indicating that much of the between-stratum variation could be explained by the addition of these fixed effects. The fixed effects in both MAIHDA models retained similar directions and magnitudes compared to the multivariable logistic regression models. The predicted probabilities for each stratum showed heterogeneity in risk of an incident HIV diagnosis for both men and women (Figs. 1 and 2), albeit risk of a new HIV diagnosis was small overall. Men aged between 25 and 49 years old, with a first generation migration background, an income below the poverty line, and who used antidepressants had the highest risk of newly diagnosed HIV (0.036% predicted risk, 95% CI = 0.025–0.052), while men aged ≥50 years with no migration background and a high income had the lowest risk (0.003% predicted risk, 95% CI = 0.003–0.004, Fig. 1; Supplementary Table S3). Similarly, women aged 25–49 years, with a first generation migration background, an income below the poverty line, who received social welfare, and who used antipsychotic medication had the highest predicted risk of newly diagnosed HIV (0.019%, 95% CI = 0.011–0.035, Fig. 2; Supplementary Table S4).

**Table 3:** Determinants of a new HIV diagnosis stratified for men and women. Results from the MAIHDA model.

	Men (72 stratum, 75,780,204 observations)				Women (72 stratum, 66,820,265 observations)			
	Basic model		Model with fixed effects		Basic model		Model with fixed effects	
	OR	95% CI	OR	95% CI	OR	95% CI	OR	95% CI
**Fixed effects: regression coefficients**								
Intercept	0.0001	0.0001–0.0001	0.00006	0.00005–0.00008	0.00003	0.0002–0.00004		
**Age**								
<25 years			REF				REF	
25–49			1.97	1.79–2.16			1.50	1.22–1.85
≥50			0.90	0.81–0.99			0.92	0.73–1.15
**Migration background**								
None			REF				REF	
First generation			1.79	1.46–2.19			3.74	2.59–4.65
Second generation			1.10	0.87–1.39			1.56	1.09–2.22
**Income**								
High			REF				REF	
Middle-low			1.02	0.81–1.28			2.87	1.97–4.17
Below the poverty line^a^			1.22	0.95–1.55			4.88	3.31–7.18
**Received social welfare**								
No			NA				REF	
Yes			NA				1.70	1.27–2.29
**Used mantal health care**^b^								
No			REF				NA	
Yes			0.97	0.80–1.18			NA	
**Used antidepressants**^c^								
No			REF				REF	
Yes			1.32	1.10–1.59			1.04	0.79–1.37
**Used anti-psychotic medication**^c^								
No			REF				REF	
Yes			0.81	0.65–1.00			1.56	1.10–2.20
**Random effects: variances**								
Stratum level	0.16	0.10–0.26	0.05	0.03–0.11	0.84	0.52–1.37	0.06	0.03–1.15
**Summary statictics**								
Variance partition coefficient (VPC)	4.6%		1.6%		20.4%		1.9%	
Proportional change in variance (PCV)			66.6%				92.5%	
Area under the receiver operating curve (AUC)	0.64		0.67		0.80		0.80	

a Income below the poverty line is defined as a household income <120% of the social minimum (the minimal amount of financial resources required to achieve a minimally acceptable lifestyle). The social minimum is determined and adjusted bi-annually by the Ministry of Social Affairs and Employment (https://www.uwv.nl/nl/toeslag/sociaal-minimum).

b Defined as declared cost (>0 euro) for mental health care.

c Use of medication for depression (ATC code N06A) or psychosis (ATC code N05A).

## Discussion

In this nationwide study comparing people newly diagnosed with HIV to the general population in the Netherlands, we quantitatively report large socio-demographic, -economic, and health related disparities in being newly diagnosed with HIV. More specifically, HIV burden was higher among men and women with a migration background, those with an low to middle income or an income below the poverty line, and those who used mental health related care of medication. We also found that these factors jointly shape risk of newly diagnosed HIV across intersectional strata. These findings allow for more concrete understanding of which subgroups could benefit from improved HIV prevention and testing.

Some of the characteristics identified as more frequent in those with HIV reflect certain demographic features of the HIV epidemic and are not entirely surprising. MSM are a key population for HIV in the Netherlands, which drives the higher proportion of men than women with HIV. Individuals younger than 25 are far less often diagnosed with HIV,^2^ reflecting the older age-distribution of those with HIV, the natural course of HIV when HIV is diagnosed late, the aging population with HIV, and the effect of preventative measures, such as treatment-as-prevention and pre-exposure prophylaxis (PrEP), on new diagnoses.^2^^,^^17^, ^18^, ^19^

Additionally, individuals newly diagnosed with HIV were more often from a first- or second-generation migration background compared to the general population, congruent with epidemiological data across Europe.^20^ As we removed individuals with a pre-migration HIV diagnosis from our analyses, this showed that HIV acquisition post-migration continues to be observed in the Netherlands and across many European countries.^20^ Results from the MAIHDA models further suggest that individuals with a first-generation migration background may belong to intersectional groups with a higher risk of newly diagnosed HIV, who could benefit from targeted HIV prevention strategies. However, we cannot determine the exact timing of HIV acquisition among individuals with a first generation migration background. It is estimated that 70% of first-generation migrants already acquired HIV pre-migration^21^ and thus HIV could not have been prevented through services available in the Netherlands.

People with HIV more often had an income below the poverty line and women with HIV received social welfare more often than the general population at the time of HIV diagnosis. Economic marginalization is known to be associated with lower HIV awareness (and HIV risk perception) and access to HIV testing^1^ and HIV prevention programs.^22^ In fact, some cities in the Netherlands have attempted to mitigate decreased access to PrEP in populations of low socioeconomic status by prioritizing their inclusion into the National PrEP program, particularly for people without insurance at increased risk of HIV acquisition.^23^ However, structural barriers (e.g., frequent visits leading to missed work days, travel costs) may further limit access to PrEP care regardless of prioritized availability.^24^ One additional and concerning finding is the disparity in low income and social welfare between individuals with and without HIV being more pronounced in women. This may indicate an intersection of vulnerabilities that complicate participation in HIV care and HIV prevention programs, particularly when combined with migration-related barriers (e.g., low-literacy, stigmatization and/or discrimination) and the challenge of navigating healthcare systems in a new setting.^20^^,^^25^ Moreover, women in general are currently not considered a key population for PrEP in the Netherlands. To optimize HIV prevention and generate targeted prevention measures, qualitative research in those with newly diagnosed HIV could help to more carefully identify the intersecting structural, social, and individual factors that place individuals at increased risk of acquiring HIV. Moreover, barriers to PrEP programs need to be decreased, for instance through online monitoring options and at home HIV and STI testing.^26^ Long-acting PrEP options could potentially also help reduce these barriers.^27^^,^^28^

It should be mentioned that after acquiring HIV, socio-economic factors (e.g., employment) can also directly and indirectly influence access to treatment, achieving viral suppression while on treatment, and risk of HIV-related morbidities and mortality.^1^^,^^5^, ^6^, ^7^^,^^20^ Even in the context of universal HIV care, socio-economic status has been shown to negatively affect treatment success in terms of viral suppression.^6^ Coupled with the findings from our study, there appear to be substantial socio-economic disparities in many facets in the public health and clinical response to HIV in the Netherlands. Part of these disparities could be owing to the longstanding structural inequalities of healthcare in general that should be addressed to provide equal care to all. There is then a clear opportunity for public health and healthcare structures in the Netherlands to broaden and facilitate their services to the underserved populations identified in this study.

One important finding from this study was that men and women with newly diagnosed HIV used mental health care more often compared to the general population. In addition, men with an incident HIV diagnosis also used antidepressants more often and women with an incident HIV diagnosis used anti-psychotic medication more often. A previous systematic review reported a relatively high prevalence of mental health problems among people with HIV, including depression, anxiety and suicidal ideation,^29^ while we also show that mental health problems may also be present before HIV diagnosis. If the onset of mental health problems and seeking mental health care services was prior to HIV diagnosis or infection, integrating HIV prevention services into mental health care would be warranted.^30^

A major strength of our study is the comprehensive prospective cohort comprising 97% of all individuals receiving HIV care in the Netherlands. Combining these data with data from Statistics Netherlands provides a unique tool for tracking sociodemographic changes in the HIV epidemic at the national level. Nevertheless, this study is not without limitations. First, 8% of the population with HIV could not be combined with data from Statistics Netherlands. People must have a postal code by the end of the calendar year to be included in the data from Statistics Netherlands, which would then more likely exclude unregistered migrants, homeless individuals, or other individuals with intersecting vulnerabilities. While we cannot determine whether HIV diagnosis or socio-economic hardship is higher in this group, they likely face greater barriers to care and social support. Their exclusion may have thus resulted in an underestimation of socio-economic disparities in people newly diagnosed with HIV in the Netherlands. Second, data on the reasons why individuals had low income or received mental health care were unavailable, making it difficult to infer on the underlying mechanisms of our findings. Third, we cannot regroup individuals to specific key subpopulations (e.g., MSM or injecting drug users), hence these results only represent the overall populations. Fourth, while associations between socio-economic factors and HIV diagnoses were identified, the temporal direction between exposure and outcome cannot be established with the cross-sectional design of our study and thus causality cannot be determined. Fifth, since we did not have the date of HIV acquisition, we were obliged to use HIV diagnoses as our outcome instead. There can be a considerable lag between these events, particularly among people who present with late stage disease, which represents a substantial portion of new diagnoses in the Netherlands. However, only mental health care consumption is likely to change over time, and other variables, such as migration background and income, are generally relatively stable, hence their association with HIV would be similar regardless of using acquisition versus diagnosis. Last, linkage between the ATHENA cohort and Statistics Netherlands was performed using a probabilistic approach with exact matching on date of birth, sex, and postal code. While this approach minimizes the risk of false matches, linkage errors could still have occurred, especially in densely populated areas, such as the Netherlands, where individuals may share identical matching characteristics.

In conclusion, a disproportionally higher burden of new HIV diagnoses was observed for individuals with not only a migration background, but also economic and mental health vulnerabilities. Barriers to HIV prevention and testing need to be reduced if we are to achieve no new HIV infections and end the HIV epidemic.

## Contributors

VJ, AB and MvdV conceptualized and designed this study. VJ and AB accessed and verified the data. VJ and AB were involved in the data management and analysis. Only authors (VJ, AB, AvS, MvdV) authorized by Statistics Netherlands had access to the data in the study. VJ, AB, and MvdV were involved with interpretation of the data. PC was added as co-author as she significantly contributed to the revision of the whole manuscript, in particular for the methodology to be used to answer reviewers. VJ drafted the manuscript. All authors revised and approved the final manuscript. MvdV had final responsibility for the decision to submit for publication.

## Data sharing statement

All results presented here are calculated from non-public registry data from Centraal Bureau voor de Statistiek (CBS), accessed through the Remote Access environment. CBS was not involved in the calculation of any of the results presented. While the data are not publicly available, academic institutions can apply for access to the Remote Access environment through the CBS (for additional information, see https://www.cbs.nl/en-gb/our-services/customised-services-microdata/microdata-conducting-your-own-research).

ATHENA cohort data (without CBS data) used in this study are available upon reasonable request. Requests for data access can be made to: hiv.monitoring@amsterdamumc.nl. Requests will be reviewed on a case-by-case basis. Microdata from Statistics Netherlands.

Statistical information or data for separate research purposes from the ATHENA cohort can be requested by submitting a research proposal to SHM (https://www.hiv-monitoring.nl/english/research/research-projects/). The proposal will undergo review by representatives of SHM for evaluation of scientific value, relevance of the study, design, and feasibility, statistical power, and overlap with existing projects.

## Declaration of interests

AB received speaker's fees from Gilead Sciences. MvdV received unrestricted research grants and consultation fees for participation in advisory boards from Gilead Sciences, MSD and ViiV, all paid to his institution. All other authors declare no competing interest.

## References

[1] World Health Organization (WHO) (2021). State of inequality: HIV, tuberculosis and malaria. https://www.who.int/data/inequality-monitor/publications/report_2021_hiv_tb_malaria.

[2] van Sighem A.I., Wit F.W.N.M., Boyd A.C., Smit C., Jongen V.W., T.S. B. Monitoring Report 2024 (2024). Human Immunodeficiency Virus (HIV) Infection in the Netherlands. https://www.hiv-monitoring.nl/nl/resources/monitoring-report-2024.

[3] Hargreaves J.R., Bonell C.P., Boler T. (2008). Systematic review exploring time trends in the association between educational attainment and risk of HIV infection in Sub-Saharan Africa. AIDS.

[4] Ward-Peterson M., Fennie K., Mauck D. (2018). Using multilevel models to evaluate the influence of contextual factors on HIV/AIDS, sexually transmitted infections, and risky sexual behavior in Sub-Saharan Africa: a systematic review. Ann Epidemiol.

[5] Menza T.W., Hixson L.K., Lipira L., Drach L. (2021). Social determinants of health and care outcomes among people with HIV in the United States. Open Forum Infect Dis.

[6] Abgrall S., del Amo J. (2016). Effect of sociodemographic factors on survival of people living with HIV. Curr Opin HIV AIDS.

[7] Probst C., Parry C.D.H., Rehm J. (2016). Socio-economic differences in HIV/AIDS mortality in South Africa. Trop Med Int Health.

[8] PHAROS (2019). Toegang tot zorg voor ongedocumenteerde migranten. https://www.pharos.nl/wp-content/uploads/2019/05/Toegang_tot_zorg_voor_ongedocumenteerde_migranten-Pharos.pdf.

[9] Heestermans T., Browne J.L., Aitken S.C., Vervoort S.C., Klipstein-Grobusch K. (2016). Determinants of adherence to antiretroviral therapy among HIV-positive adults in Sub-Saharan Africa: a systematic review. BMJ Glob Health.

[10] Biswas D., Toebes B., Hjern A., Ascher H., Norredam M. (2012). Access to health care for undocumented migrants from a human rights perspective: a comparative study of Denmark, Sweden, and the Netherlands. Health Hum Rights.

[11] Bil J.P., Zuure F.R., Alvarez-del Arco D. (2019). Disparities in access to and use of HIV-related health services in the Netherlands by migrant status and sexual orientation: a cross-sectional study among people recently diagnosed with HIV infection. BMC Infect Dis.

[12] Marmot M., Allen J., Goldblatt P., Herd E., Morrison J. (2020). Build back fairer: the COVID-19 marmot review. The pandemic, socioeconomic and health inequalities in England. https://www.instituteofhealthequity.org/resources-reports/build-back-fairer-the-covid-19-marmot-review.

[13] Homan P. (2019). Structural sexism and health in the United States: a new perspective on health inequality and the gender system. Am Sociol Rev.

[14] Boender T.S., Smit C., Sighem A.V. (2018). AIDS therapy evaluation in the Netherlands (ATHENA) national observational HIV cohort: cohort profile. BMJ Open.

[15] Uitvoeringsinstituut Werknemersverzekeringen (UWV) (2024). Sociaal minimum. https://www.uwv.nl/nl/toeslag/sociaal-minimum.

[16] Evans C.R., Leckie G., Subramanian S.V., Bell A., Merlo J. (2024). A tutorial for conducting intersectional multilevel analysis of individual heterogeneity and discriminatory accuracy (MAIHDA). SSM Popul Health.

[17] Smit M., Brinkman K., Geerlings S. (2015). Future challenges for clinical care of an ageing population infected with HIV: a modelling study. Lancet Infect Dis.

[18] Medland N.A., McManus H., Bavinton B.R. (2024). HIV incidence in people receiving government-subsidised pre-exposure prophylaxis in Australia: a whole-of-population retrospective cohort study. Lancet HIV.

[19] Cohen M.S., Chen Y.Q., McCauley M. (2016). Antiretroviral therapy for the prevention of HIV-1 transmission. N Engl J Med.

[20] Nöstlinger C., Cosaert T., Landeghem E.V. (2022). HIV among migrants in precarious circumstances in the EU and European economic area. Lancet HIV.

[21] Mann S., Mougammadou Z., Wohlfahrt J., Elmahdi R. (2024). Post-migration HIV acquisition: a systematic review and meta-analysis. Epidemiol Infect.

[22] Hojilla J.C., Hurley L.B., Marcus J.L. (2021). Characterization of HIV preexposure prophylaxis use behaviors and HIV incidence among US adults in an integrated health care system. JAMA Netw Open.

[23] Wijstma E., Jongen V.W., Boyd A. (2025). Outcomes of a policy to prioritize populations with expected healthcare barriers for subsidized preexposure prophylaxis care in Amsterdam, the Netherlands. AIDS.

[24] Twisk D.E., Meima B., Nieboer D., Richardus J.H., Götz H.M. (2021). Distance as explanatory factor for sexual health centre utilization: an urban population-based study in the Netherlands. Eur J Publ Health.

[25] Segala F.V., Di Gennaro F., Frallonardo L. (2024). HIV-related outcomes among migrants living in Europe compared with the general population: a systematic review and meta-analysis. Lancet HIV.

[26] Hanneke Goense C.J.D., Evers Y.J., Hoebe C., Dukers-Muijrers N. (2025). A perspective on home-based sexual health care: evidence, access, and future directions. Curr HIV AIDS Rep.

[27] Kelley C.F., Acevedo-Quinones M., Agwu A.L. (2024). Twice-yearly lenacapavir for HIV prevention in men and gender-diverse persons. N Engl J Med.

[28] Bekker L.G., Das M., Abdool Karim Q. (2024). Twice-yearly lenacapavir or daily F/TAF for HIV prevention in cisgender women. N Engl J Med.

[29] Yang Y., Chen B., Zhang H. (2024). Global prevalence of depressive symptoms among people living with HIV/AIDS: a systematic review and meta-analysis of the past five years. AIDS Care.

[30] Remien R.H., Stirratt M.J., Nguyen N., Robbins R.N., Pala A.N., Mellins C.A. (2019). Mental health and HIV/AIDS: the need for an integrated response. AIDS.

