# Supplementary material for: Risk of new HIV diagnosis by intersecting migration, socioeconomic, and mental health vulnerabilities in the Netherlands: a nationwide analysis of the ATHENA cohort and Statistics Netherlands registry data
Source: Lancet Reg Health Eur. 2025 Nov 20;60:101508. doi: 10.1016/j.lanepe.2025.101508 (PMC12765169; doi:10.1016/j.lanepe.2025.101508)
Supplement: Supplementary Tables [file mmc1.pdf]

Supplement to
Investigating social inequalities in people with a new HIV diagnosis in the Netherlands: results from the ATHENA national observational cohort and Statistics Netherlands

Vita W. Jongen, Anders Boyd, Patrizia Carrieri, Nina Schat, Selwyn H. Lowe, Rosan van Zoest, Marit G.A. van Vonderen, Jolanda Lammers, Mark Verhagen, Ard van Sighem, Marc van der Valk, on behalf of the ATHENA observational HIV cohort

Supplementary Table 1. Determinants of an HIV diagnosis. Results from univariable logistic regression. 2

Supplementary Table 2. Distribution of socio-demographic and socio-economic factors among women with and without missing income data 3

Supplementary Table 3. Strata with the highest and lowest risk of a new HIV diagnoses for men. 4

Supplementary Table 4. Strata with the highest and lowest risk of a new HIV diagnoses for women.......... 5

Appendix A. Collaborators of the ATHENA observational HIV cohort 7

Supplementary Table 1. Determinants of an HIV diagnosis. Results from univariable logistic regression.

	Men		Women	
	OR	*95% CI*	*OR*	*95% CI*
**Migration background**				
None	REF		REF	
First generation	2·57	2·43-2·71	6·77	5·93-7·74
Second generation	1·4	1·28-1·52	1·96	1·56-2·46
**Income**				
High	REF		REF	
Middle-low	1·38	1·30-1·46	3·06	2·53-3·70
Below the poverty line[1]	2·39	2·23-2·56	9·50	7·86-11·48
**Received social welfare**				
No	REF		REF	
Yes	1·96	1·79-2·15	4·6	3·95-5·36
**Used mental health care[2]**				
No	REF		REF	
Yes	1·37	1·24-1·51	1·16	0·93-1·44
**Used antidepressants[3]**				
No	REF		REF	
Yes	1·77	1·62-1·94	1·44	1·19-1·74
**Used anti-psychotic medication[3]**				
No	REF		REF	
Yes	1·33	1·14-1·56	2·58	1·94-3·44

1· Income below the poverty line is defined as a household income <120% of the social minimum (the minimal amount of financial resources required to achieve a minimally acceptable lifestyle). The social minimum is determined and adjusted bi-annually by the Ministry of Social Affairs and Employment (https://www.uwv.nl/nl/toeslag/sociaal-minimum).
2. Defined as declared cost (>0 euro) for mental health care
3. Use of medication for depression (ATC code N06A) or psychosis (ATC code N05A)

Supplementary Table 2. Distribution of socio-demographic and socio-economic factors among women with and without missing income data

	Men		Women	
**Sample size per stratum**	**Number of strata**	**% of strata**	**Number of strata**	**% of strata**
1,000,000 or more	14	6·5%	14	6·5%
100,000 or more	41	19·0%	47	21·8%
10,000 or more	128	59·3%	111	51·4%
1000 or more	201	93·1%	177	82·0%
500 or more	209	96·8%%	186	86·1%
100 or more	215	99·5%	208	96·3%
50 or more	216	100%%	212	98·2%
30 or more	216	100%%	214	99·1%
10 or more	216	100%%	215	99·5%
less than 10	0	0%	1	0·5%

Supplementary Table 3. Strata with the highest and lowest risk of a new HIV diagnoses for men.

Age	Migration background	Income[1]	Mental health care use	Use of antidepressants	Use of anti-psychotic medication	Sample size	Predicted risk	95%CI
	**Highest risk**							
25-49 years	1st generation	Below the poverty line	No	Yes	No	48,704	0·036	0·025-0·052
25-49 years	1st generation	Low-middle	No	Yes	No	64,767	0·035	0·024-0·049
25-49 years	1st generation	High	No	Yes	No	29,357	0·034	0·023-0·052
25-49 years	1st generation	Low-middle	Yes	Yes	No	24,122	0·032	0·021-0·050
25-49 years	1st generation	Below the poverty line	Yes	Yes	No	21,442	0·031	0·019-0·049
	**Lowest risk**							
≥50 years	2nd generation	High	No	No	No	776,745	0·004	0·003-0·006
≥50 years	No migration background	Low-middle	No	No	No	11,717,392	0·004	0·003-0·005
≥50 years	No migration background	High	Yes	No	Yes	18,464	0·004	0·002-0·007
<25 years	No migration background	High	No	No	No	3,542,066	0·003	0·003-0·004
≥50 years	No migration backgroynd	High	No	No	No	12,134,635	0·003	0·002-0·004

1. Income below the poverty line is defined as a household income <120% of the social minimum (the minimal amount of financial resources required to achieve a minimally acceptable lifestyle). The social minimum is determined and adjusted bi-annually by the Ministry of Social Affairs and Employment (https://www.uwv.nl/nl/toeslag/sociaal-minimum).

Supplementary Table 4. Strata with the highest and lowest risk of a new HIV diagnoses for women.

Age	Migration background	Income[1]	Received social welfare	Use of antidepressants	Use of anti-psychotic medication	Sample size	Predicted risk	95%CI
	**Highest risk**							
25-49 years	1st generation	Below the poverty line	Yes	No	Yes	14,577	0·019	0·011-0·035
25-49 years	1st generation	Below the poverty line	Yes	Yes	Yes	19,408	0·019	0·011-0·034
25-49 years	1st generation	Below the poverty line	No	No	Yes	11,525	0·014	0·007-0·025
25-49 years	1st generation	Below the poverty line	No	Yes	Yes	13,451	0·013	0·007-0·024
25-49 years	1st generation	Below the poverty line	Yes	Yes	No	74,389	0·013	0·008-0·021
	**Lowest risk**							
≥50 years	No migration background	High	No	Yes	No	850,512	0·0004	0·0002-0·0006
<25 years	2nd generation	High	No	No	No	381,206	0·0005	0·0002-0·0005
≥50 years	2nd generation	High	No	No	No	576,867	0·0003	0·0002-0·0006
<25 years	No migration background	High	No	No	No	2,846,477	0·0003	0·0002-0·0004
≥50 years	No migration background	High	No	No	No	8,778,458	0·0002	0·0002-0·0004

1. Income below the poverty line is defined as a household income <120% of the social minimum (the minimal amount of financial resources required to achieve a minimally acceptable lifestyle). The social minimum is determined and adjusted bi-annually by the Ministry of Social Affairs and Employment (https://www.uwv.nl/nl/toeslag/sociaal-minimum).

Appendix A. Collaborators of the ATHENA observational HIV cohort

Clinical centres

** denotes site coordinating physician*

Amsterdam UMC, Amsterdam:
HIV treating physicians: F.J.B. Nellen*, M.A. van Agtmael, M. Bomers, G.J. de Bree, S.E. Geerlings, A. Goorhuis, V.C. Harris, J.W. Hovius, B. Lemkes, E.J.G. Peters, T. van der Poll, J.M. Prins, K.C.E. Sigaloff, V. Spoorenberg, M. van der Valk, M. van Vugt, W.J. Wiersinga, F.W.M.N. Wit. *HIV nurse consultants*: C. Bruins, J. van Eden, I.J. Hylkema-van den Bout, L.M. Laan, F.J.J. Pijnappel, S.Y. Smalhout, M.E. Spelbrink, A.M. Weijsenfeld. *HIV clinical virologists/chemists:* N.K.T. Back, R. van Houdt, M. Jonges, S. Jurriaans, F. van someren Gréve, M.R.A. Welkers, K.C. Wolthers.

Emma Kinderziekenhuis (Amsterdam UMC), Amsterdam:
HIV treating physicians: M. van der Kuip, D. Pajkrt. *HIV nurse consultants*: F.M. Hessing, A.M. Weijsenfeld.

Admiraal De Ruyter Ziekenhuis, Goes:
HIV treating physicians: M. van den Berge*, A. Stegeman. *HIV nurse consultants*: S. Baas, L. Hage de Looff. *HIV clinical virologists/chemists*: A. van Arkel, J. Stohr, B. Wintermans.

Catharina Ziekenhuis, Eindhoven:
HIV treating physicians: M.J.H. Pronk*, H.S.M. Ammerlaan. *HIV nurse consultants*: E.S. de Munnik, S. Phaf. *HIV clinical virologists/chemists*: B. Deiman, V. Scharnhorst, M.C.A. Wegdam.

DC Klinieken Lairesse – Hiv Focus Centrum, Amsterdam:
HIV treating physicians: J. Nellen*, A. van Eeden, E. Hoornenborg, S de Stoppelaar.
HIV nurse consultants: H. Berends, L.J.M. Elsenburg, H. Nobel. *HIV clinical virologists/chemists*: F. van Someren Gréve, M. Welkers, K. Wolthers, N. Back, S. Jurriaans

ETZ (Elisabeth-TweeSteden Ziekenhuis), Tilburg:
HIV treating physicians: M.E.E. van Kasteren*, M.A.H. Berrevoets, A.E. Brouwer. *HIV nurse specialist*: A. Adams, B.A.F.M. de Kruijf-van de Wiel. *HIV nurse consultants*: M. Pauwels-van Rijkevoorsel. *HIV data collection*: B.A.F.M. de Kruijf-van de Wiel. *HIV clinical virologists/chemists*: J.L. Murck.

Erasmus MC, Rotterdam:
HIV treating physicians: C. Rokx*, A.A. Anas, H.I. Bax, E.C.M. van Gorp, M. de Mendonça Melo, E. van Nood, J.L. Nouwen, B.J.A. Rijnders, C.A.M. Schurink, L. Slobbe, T.E.M.S. de Vries-Sluijs. *HIV nurse consultants*: N. Bassant, J.E.A. van Beek, M. Vriesde, L.M. van Zonneveld. *HIV data collection*: J. de Groot. *HIV clinical virologists/chemists*: J.J.A. van Kampen, M.P.G Koopmans.

Erasmus MC Sophia Kinderziekenhuis, Rotterdam:
HIV treating physicians: P.L.A. Fraaij, A.M.C. van Rossum, C.L. Vermont. *HIV nurse consultants*: L.C. van der Knaap.

Flevoziekenhuis, Almere:
HIV treating physicians: J. Branger*, R.A. Douma. *HIV nurse consultant*: A.S. Cents-Bosma, M.A. Mulder.

HagaZiekenhuis, Den Haag:
HIV treating physicians: E.F. Schippers*, C. de Bree, C. van Nieuwkoop. *HIV nurse consultants*: J. Geilings, A. van Overeem. *HIV data collection*: G. van der Hut. *HIV clinical virologists/chemists*: N.D. van Burgel.

HMC (Haaglanden Medisch Centrum), Den Haag:
HIV treating physicians: E.M.S. Leyten*, L.B.S. Gelinck, F. Mollema. *HIV nurse consultants*: M. Langbein, G.S. Wildenbeest. *HIV clinical virologists/chemists*: T. Nguyen.

Isala, Zwolle:
HIV treating physicians: B. Hafkamp*, J.W. Bouwhuis, A.J.J. Lammers. *HIV nurse consultants*: A.G.W. van Hulzen, S. Kraan. *HIV clinical virologists/chemists*: S.B. Debast, G.H.J. Wagenvoort.

Leids Universitair Medisch Centrum, Leiden:

HIV treating physicians:
A.H.E. Roukens*, M.G.J. de Boer, H. Jolink, M.M.C. Lambregts, H. Scheper.
HIV nurse consultants: A. Metselaar, D. van der Sluis. *HIV clinical virologists/chemists*: S.A. Boers, E.C.J. Claas, E. Wessels.

Maasstad Ziekenhuis, Rotterdam:
HIV treating physicians: J.G. den Hollander*, R. El Moussaoui, K. Pogany. *HIV nurse consultants*: C.J. Brouwer, D. Heida-Peters, E. Mulder, J.V. Smit, D. Struik-Kalkman.
HIV data collection: T. van Niekerk. HIV clinical virologists/chemists: C. van Tienen.

Maastricht UMC+, Maastricht:
HIV treating physicians: S.H. Lowe*, A.M.L. Oude Lashof, D. Posthouwer, A. Stoop, M.E. van Wolfswinkel.
HIV nurse consultants: R.P. Ackens, M. Elasri, K. Houben-Pintaric, J. Schippers.
HIV clinical virologists/chemists/pharmacist: T.R.A. Havenith, I.H.M. van Loo.

Frisius MC, Leeuwarden:
HIV treating physicians: M.G.A. van Vonderen*, L.M. Kampschreur, S.E. van Roeden. *HIV nurse consultants*: M.C. van Broekhuizen, S. Faber *HIV clinical virologists/chemists*: A. Al Moujahid.

Medisch Spectrum Twente, Enschede:
HIV treating physicians: G.J. Kootstra*, C.E. Delsing. *HIV nurse consultants*: M. van der Burg-van de Plas, L. Scheiberlich.

Noordwest Ziekenhuisgroep, Alkmaar:
HIV treating physicians: W. Kortmann*, G. van Twillert*, R. Renckens, J. Wagenaar.
HIV nurse consultants & HIV data collection: D. Ruiter-Pronk, B. Stander.
HIV clinical virologists/chemists: J.W.T. Cohen Stuart, M. Hoogewerf, W. Rozemeijer, J.C. Sinnige.

OLVG, Amsterdam:
HIV treating physicians: K. Brinkman*, G.E.L. van den Berk, K.D. Lettinga, M. de Regt, W.E.M. Schouten, J.E. Stalenhoef, S.M.E. Vrouenraets. *HIV nurse consultants*: H. Blaauw, G.F. Geerders, M.J. Kleene, M. Knapen, M. Kok, I.B. van der Meché, A.J.M. Toonen, S. Wijnands, E. Wttewaal. *HIV clinical virologists*: D. Kwa, T.J.W. van de Laar.

Radboudumc, Nijmegen:
HIV treating physicians: R. van Crevel*, K. van Aerde, R.J.W. Arts, S.S.V. Henriet, H.J.M. ter Hofstede, J. Hoogerwerf, O. Richel, K. Stol. *HIV nurse consultants*: M. Albers, K.J.T. Grintjes-Huisman, M. de Haan, M. Marneef. *HIV clinical virologists/chemists*: M. McCall, J. Rahamat-Langendoen, E. Ruizendaal. *HIV clinical pharmacology consultant*: D. Burger.

Rijnstate, Arnhem: *HIV treating physicians:* E.H. Gisolf*, M. Claassen, R.J. Hassing,. *HIV nurse consultants:* G. ter Beest, P.H.M. van Bentum, Y. Neijland, M. Valette. *HIV clinical virologists/chemists:* C.M.A. Swanink, M. Klein Velderman.

Spaarne Gasthuis, Haarlem:
HIV treating physicians: S.F.L. van Lelyveld*, R. Soetekouw. *HIV nurse consultants*: L.M.M. van der Prijt, J. van der Swaluw. *HIV clinical virologists/chemists*: J.S. Kalpoe, A. Wagemakers, A. Vahidnia.

Medisch Centrum Jan van Goyen, Amsterdam:
HIV treating physicians: F.N. Lauw, D.W.M. Verhagen. *HIV nurse consultants*: M. van Wijk.

Universitair Medisch Centrum Groningen, Groningen:
HIV treating physicians: W.F.W. Bierman*, M. Bakker, J. Kleinnijenhuis, E. Kloeze, A. Middel, D.F. Postma, Y. Stienstra, M. Wouthuyzen-Bakker. *HIV nurse consultants*: A. Boonstra, M.M.M. Maerman, D.A. de Weerd. *HIV clinical virologists/chemists*: M. Knoester, C.C. van Leer-Buter, H.G.M. Niesters, X.W. Zhou.

Beatrix Kinderziekenhuis (Universitair Medisch Centrum Groningen), Groningen:
HIV treating physicians: B.R. Brandsema, A.R. Verhage. *HIV nurse consultants:* N. van der Woude. *HIV clinical virologists/chemists*: M. Knoester, C.C. van Leer-Buter, H.G.M. Niesters, X.W. Zhou.

Universitair Medisch Centrum Utrecht, Utrecht:
HIV treating physicians: T. Mudrikova*, R.E. Barth, A.H.W. Bruns, P.M. Ellerbroek, M.P.M. Hensgens, J.J. Oosterheert, E.M. Schadd, A. Verbon, B.J. van Welzen. *HIV nurse consultants*: B.M.G. Griffioen-van Santen, L. van de Koolwijk, I. de Kroon. *HIV clinical virologists/chemists*: F.M. Verduyn Lunel, A.M.J. Wensing.

Wilhelmina Kinderziekenhuis, UMC Utrecht, Utrecht:
HIV treating physicians: Y.G.T. Loeffen, T.F.W. Wolfs. *HIV nurse consultants*: M. Kok. *HIV clinical virologists/chemists*: F.M. Verduyn Lunel, A.M.J. Wensing.

Curaçao Medical Center, Willemstad (Curaçao): *HIV treating physicians*: E.O.W. Rooijakkers, D. van de Wetering. *HIV nurse consultants*: A. Alberto. *Data collection*: I. der Meer.

Coordinating center
Board of directors: M. van der Valk, S. Zaheri.
HIV data analysis: A.C. Boyd, D.O. Bezemer, V.W. Jongen, A.I. van Sighem, C. Smit, F.W.M.N. Wit.
Data HIV data management and quality control: M.M.J. Hillebregt, T.J. Woudstra, T. Rutkens
HIV data monitoring: D. Bergsma, J.M. Grolleman, L.E. Koster, K.J. Lelivelt, S.T. van Loenen, M.J.C. Schoorl, K.M. Visser.
HIV data collection: K.J. Lelivelt, K.M. Visser, M. van den Akker, O.M. Akpomukai, R. Alexander, Y.M. Bakker, L. Bastos Sales, A. el Berkaoui, M. Bezemer-Goedhart, C.B.J. Bon, E.A. Djoechro, I. el Hammoud, M.R. Khouw, C.R.E. Lodewijk, E.G.A. Lucas, S. van Meerveld-Derks, M.A. van Montfoort, H.W. Mulder, L. Munjishvili, C.M.J. Ree, R. Regtop, A.F. van Rijk, Y.M.C. Ruijs-Tiggelman, P.P. Schnörr, R. van Veen, W.H.G. van Vliet-Klein Gunnewiek, E.C.M Witte.
Patiënt registration: D. Bergsma, Y.M.C. Ruijs-Tiggelman.
